# SARS-CoV-2 Infection in Children in Southern Italy: A Descriptive Case Series

**DOI:** 10.3390/ijerph17176080

**Published:** 2020-08-21

**Authors:** Daniela Loconsole, Desirèe Caselli, Francesca Centrone, Caterina Morcavallo, Silvia Campanella, Maurizio Aricò, Maria Chironna

**Affiliations:** 1 Department of Biomedical Sciences and Human Oncology-Hygiene Section, University of Bari, Piazza G. Cesare 11, 70124 Bari, Italy; daniela.loconsole@uniba.it (D.L.); francesca.centrone@uniba.it (F.C.); caterina.morcavallo@uniba.it (C.M.); campanella.silvia@libero.it (S.C.); 2 Pediatric Infectious Diseases, Giovanni XXIII Children Hospital, Azienda Ospedaliero Universitaria Consorziale Policlinico, Piazza G. Cesare 11, 70124 Bari, Italy; desiree.caselli@policlinico.ba.it; 3 COVID Emergency Task Force, Giovanni XXIII Children Hospital, Azienda Ospedaliero Universitaria Consorziale Policlinico, Piazza G. Cesare 11, 70124 Bari, Italy; maurizio.arico@policlinico.ba.it

**Keywords:** COVID-19, children, SARS-CoV-2, epidemiology, surveillance, health policy, school closure, southern Italy, household transmission, lockdown

## Abstract

At the beginning of the coronavirus-2019 (COVID-19) pandemic, Italy was one of the most affected countries in Europe. Severe acute respiratory syndrome coronavirus-2 (SARS-CoV-2) infection is less frequent and less severe in children than in adults. This study analyzed the frequency of SARS-CoV-2 infection among all children aged <18 years in the Apulia region of southern Italy and the characteristics of the infected children. Clinical and demographic data were collected through the national platform for COVID-19 surveillance. Of the 166 infected children in the Apulia region, 104 (62.6%) were asymptomatic, 37 (22.3%) had mild infections, 22 (13.3%) had moderate infections, and 3 (1.8%) had severe infections. Only ten children (6.0%) were hospitalized, but none required intensive care support and none died. SARS-CoV-2 infection was transmitted mainly from parents or relatives to children. Because of school closure during the lockdown, infection was unlikely to have been transmitted among children. It is unclear whether school reopening would enhance virus spread, leading the Italian government to develop guidelines for safe school reopening. The actual role of children in virus transmission remains unclear. A sensitive surveillance system, prompt identification of cases, testing, and contact tracing will be key to reducing the further spread of infection.

## 1. Introduction

Italy was one of the most affected European countries during the first months of the coronavirus 2019 (COVID-19) pandemic, and the first to institute a national lockdown to contain the spread of the virus, effective 9 March 2020 [1]. School closure was decided some days before the beginning of the lockdown and started from 5 March 2020. As of 16 June 2020, the pandemic has resulted in 238,082 cases and 33,209 deaths in Italy [2].

Severe acute respiratory syndrome coronavirus-2 (SARS-CoV-2) infection is less frequent and less severe in children than in adults [3,4]. In addition to fever and cough, gastrointestinal symptoms have been observed in children, including vomiting, abdominal pain, and diarrhea [5]. The prognosis is almost invariably good and recovery is achieved usually within 1 or 2 weeks [6]. The reason for this advantage is not yet clear, although epithelial shedding of angiotensin-converting enzyme-2 (ACE-2), the binding site of the virus, is reduced in children, likely because of progressive maturation during childhood [7].

Overall, 4801 cases of SARS-CoV-2 infection in pediatric subjects, aged <18 years, have been registered to date in Italy, accounting for 2.0% of all registered cases. Of these children, 12.7% were aged 0–1 year, 18.0% were aged 2–6 years, and 69.4% were aged 7–17 years [2]. Consistent with its reduced severity in children, only 2.8% of diagnosed children were hospitalized, with 32.4% of these being aged 0–1 year [2].

Apulia is a large region in Southern Italy, with about four million inhabitants, including 652,754 children. The proportion of inhabitants diagnosed with COVID-19 has been lower in Apulia than in regions of northern Italy. As of 1 June 2020, 4498 cases of confirmed SARS-CoV-2 infections were reported in Apulia [8]. The first case was identified on 26 February 2020, in a 44-year-old man in Taranto province after arrival from a known COVID-19 hotspot (Lodi, Lombardy, Northern Italy).

This study analyzed the frequency of SARS-CoV-2 infection among all children and adolescents, aged <18 years, in the Apulia region and the characteristics of children and adolescents found to be positive for SARS-CoV-2.

## 2. Materials and Methods

The COVID-19 national surveillance system in Italy is an integrated microbiological and epidemiological surveillance system, which continuously and systematically collects, compares, and analyzes information on all cases of SARS-CoV-2 infection confirmed by molecular diagnostics at regional reference laboratories (Circular n. 1997 of 22 January 2020, Ministry of Health). The COVID-19 surveillance system is coordinated by the Department of Infectious Diseases at the Istituto Superiore di Sanità. Each region of Italy has one or more local coordinators. In Apulia, surveillance activities were performed by the Regional Epidemiological Observatory and the Laboratory of Molecular Epidemiology and Public Health of the Hygiene Unit (A.O.U.C. Policlinico Bari), which is the coordinator of the Regional Laboratory Network for SARS-CoV-2 diagnosis. The network consists of 12 laboratories located throughout the entire region, which consists of six provinces. There are 203,302 children residing in the province of Bari, 60,570 in the province of Brindisi, 68,151 in the province of BAT, 106,120 in the province of Foggia, 121,391 in the province of Lecce, and 93,220 in the province of Taranto. Respiratory samples (nasal and pharyngeal swabs, endotracheal aspirates, and bronchoalveolar lavage fluid) were collected from all suspected cases and their contacts, and analyzed by the Regional Network for SARS-CoV-2 diagnosis using various commercial real-time PCR (RT-PCR) kits approved by the Italian Ministry of Health [9]. The clinical and demographic information of all patients who tested positive was placed on the online surveillance platform (https://covid-19.iss.it/login.aspx?ReturnUrl=%2f). Data were collected during the period from 1 March to 1 June, and analyzed with STATA 12.0 software (StataCorp LLC, Texas 77845-4512, USA). Clinical cases were classified according to the Italian COVID-19 surveillance system (https://covid-19.iss.it/) as asymptomatic in absence of symptoms, mild infection in presence of fever <38 °C, cough, malaise, gastrointestinal symptoms without fever, moderate infection in presence of fever ≥38 °C, upper respiratory symptoms, anosmia/ageusia, gastrointestinal symptoms, and severe infection in the presence of fever ≥38 °C with dyspnea and lower respiratory tract infection.

## 3. Results

Of the 4498 confirmed cases of SARS-CoV-2 infection diagnosed in Apulia, 166 (3.7%) were in children aged <18 years, with the first one being identified on March 7 in an asymptomatic 10-year old in the province of Bari. The patients included 81 (48.8%) boys and 85 (51.2%) girls, with a median age of 11 years (interquartile range (IQR) 5–14 years). Of the 166 patients, nine (5.4%) had an associated co-morbidity, including three with cancer, four with a congenital or perinatal disease, one with asthma, and one with a congenital disease plus asthma. The demographic and clinical characteristics of these SARS-CoV-2 infected children by age groups are summarized in Table 1. At the time of diagnosis, 104 children (62.6%) were asymptomatic, 37 (22.3%) had a mild infection, and 22 (13.3%) had a moderate infection.

Only ten children (6.0%) were hospitalized, for an average of 18.2 days (range, 2–63 days). One child (0.6%), aged 3 years and already hospitalized for acute lymphoblastic leukemia (ALL), showed a severe infection and required hospitalization for 63 days. Two siblings aged 5 and 14 years, without pre-existing medical conditions and asymptomatic at diagnosis, developed severe infections within a few days, showing fever ≥38 °C and lower respiratory tract infection. They were hospitalized for 26 and 35 days, respectively, and released following complete recovery. None of the children developed critical conditions requiring admission to an intensive care unit (ICU), and none died of COVID-19.

The modality of exposure to SARS-CoV-2 was traced in 99 (59.6%) children. Except for the patient with ALL, whose infection appeared to have been acquired during hospitalization and was thus classified as nosocomial, the other 98 reported close contact with an infected individual.

**Figure 1:**
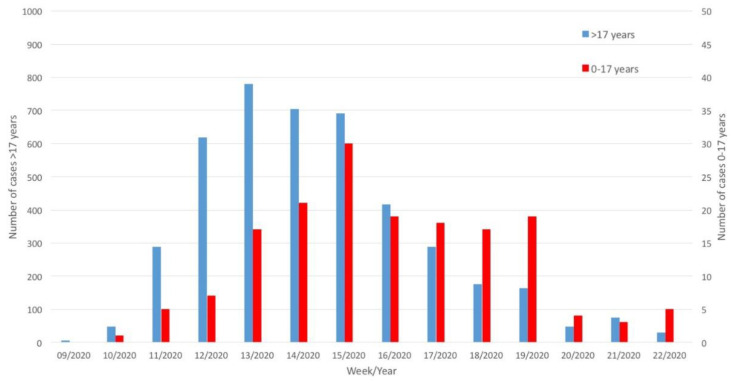
Distribution of SARS-CoV-2 cases by week (children 0–17 years vs. adults >17 years), Apulia region, Southern Italy.

A comparison of the patterns of COVID-19 diffusion among adults and children showed that the peak in children occurred two weeks after the peak in adults (Figure 1).

**Figure 2:**
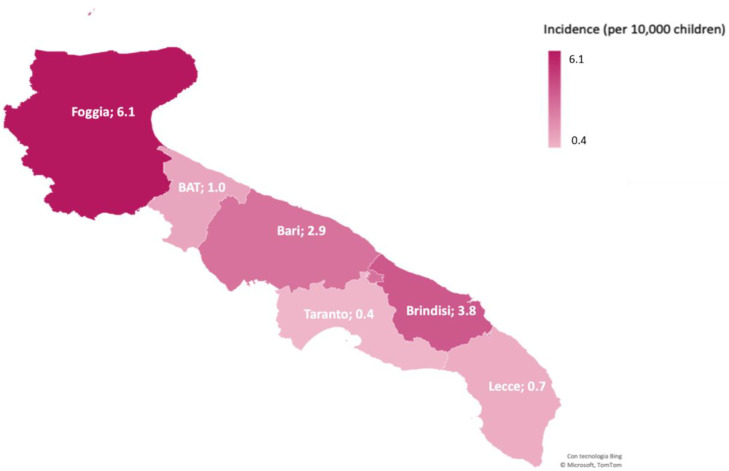
Incidence of SARS-CoV-2 infection by province in children and adolescents <18 years, Apulia region, Southern Italy.

Of the 166 infected children, 58 were registered in the province of Bari, 23 in Brindisi, seven in BAT, 65 in Foggia, nine in Lecce, and four in Taranto. The highest incidence was in the province of Foggia (6.1/10,000 children), followed by Brindisi (3.8/10,000 children) (Figure 2). Foggia was the province in Apulia with both the highest overall incidence and the highest incidence among children (data not shown). Several outbreaks occurred in this province, mostly among households.

## 4. Discussion

The ideal method of determining the incidence and prevalence of an infectious disease would be to formally screen the general population. This has been almost impossible in most situations, due to the large numbers of individuals and difficulties screening everyone. Universal screening for SARS-CoV-2 infection in a defined population is exceptional. One example is the screening of the population of Vo’, a small town in the Veneto region of Italy, by local health authorities [10]. Contact tracing of newly infected persons and reconstruction of the train of transmission revealed that most patients were infected before the lockdown or by an asymptomatic infected person living in the same household. No infections were detected in the 234 tested children aged 0–10 years, despite some of them living in the same household as infected people.

Conversely, assessments of infection most frequently involve patients evaluated at one or more hospitals. In the Coronavirus Infection in Pediatric Emergency Departments (CONFIDENCE) study, most of the infants presented with mild disease [11]. Of the infected patients, 38% were admitted to the hospital because of symptoms, irrespective of the severity of disease. Severe and critical cases were diagnosed in patients with coexisting conditions, but no deaths were reported. In another recent European report, 8% of children required ICU admission, and 4% required mechanical ventilation. Multivariate analyses showed that risk factors significantly associated with the need for ICU admission include age <1-month, male sex, comorbidities, and signs or symptoms of lower respiratory tract infection at presentation. The reported fatality rate was 0.69% [12].

The present report describes the results of a different approach. Universal screening of the over 4 million inhabitants of the Apulian region was not feasible. However, we were able to analyze data of all subjects in this region who tested positive for SARS-CoV-2 infection, particularly characteristics of children and adolescents. The most important finding was that 62% of the 166 Apulian children and adolescents positive for SARS-CoV-2 were asymptomatic; rates were higher than the 21% and 16% reported in other studies [11,12]. Moreover, contact tracing in 99 of these children showed that 98 acquired the infection from a family member. These findings indicate that in Apulia, SARS-CoV-2 infection was transmitted from parents or relatives to children, with about two-thirds of the latter not developing symptoms. However, it should be taken into account that due to social lockdown, children were only in contact with their relatives, preventing contact with people outside their family circle.

This study also found that pediatric COVID-19 did not represent a major clinical problem for the public health system, since none of the 166 infected subjects died or required ICU admission. Only 10 children (6.0%) were hospitalized, and only three (1.8%) developed severe disease. Nevertheless, a large amount of work was required to monitor all the pediatric patients with suspected COVID-19, evaluate them periodically in the referred hospital, and screen for SARS-CoV-2, usually with negative results.

Children with co-morbidities have been predicted to be at higher risk for fatal outcome or severe disease. In our region, fewer than 6% of SARS-CoV-2 infected children reported one or more chronic diseases. In contrast, a study in a large European hospital cohort found that 25% of infected children reported one or more chronic diseases [12], with a much higher hospitalization rate (62%). The 10 children hospitalized in the present study were hospitalized due to precautions, not because of actual clinical manifestations.

The epidemic curve of SARS-COV-2 infection in children in the Apulia region showed a peak two weeks later than that in adults. Although data were not available for all children, these findings are consistent with the major role played by household exposure in the transmission of SARS-CoV-2 to children. The latter is also supported by the finding that the number of infections in children remained unchanged after the lockdown.

The findings of our study do not consent to speculate on the possible scenario after school reopening. Moreover, because children and adolescents are unlikely to have been the primary source of infection, it is unclear whether school closure enhanced infection control significantly. In Italy, subjects aged <18-years are thought to account for <2% of all COVID-19 cases [13]. However, because of school closure during the lockdown, we cannot evaluate the actual role of children in the transmission of the infection. The Italian government is developing guidelines for school reopening, based on small classes, social distancing, daily monitoring of temperature, sanitization of environments, and ventilation of rooms [14]. Ideally, school reopening should not be further delayed for social and economic reasons, as well as the negative impact of school closing on children, especially those aged 2–10 years [14]. Social life at school helps in the development of personality and a sense of identity, whereas isolation could enhance the risks of anxiety, depression, and post-traumatic stress disorder [15]. A sensitive surveillance system, prompt identification of cases, testing, and contact tracing will be the keys to a safe reopening of schools.

## 5. Conclusions

Data on SARS-CoV-2 infected children and adolescents in the Apulia region of Italy confirmed that children were less affected by the current pandemic, with lower rates of infection and less severe infection. Household contacts were the most frequent source of infection, with little transmission between children and minimal clinical manifestations, with the large majority of cases remaining asymptomatic. Thus, based on current knowledge, adults, not children, should be the primary targets for strict social distancing. Rapid clearance of the virus in infected subjects is also associated with a favorable outcome and a limited impact on the surrounding community. Further studies are needed to better understand some accompanying phenomena, such as the excess of Kawasaki-like [16] or chilblain-like [17] vasculitis associated with the spread of COVID-19, although these conditions may not be associated with SARS-COV-2 infection itself. Further understanding of these phenomena will likely contribute to determining the as yet unknown mechanism of immune response during viral infections.

## Figures and Tables

**Table 1:** Demographic and clinical characteristics of SARS-CoV-2 positive children and adolescents by age groups, Apulia region, Southern Italy.

	Age Groups (Years)				
	0–1	2–6	7–11	12–17	Tot
	N. (%)	N. (%)	N. (%)	N. (%)	N. (%)
Cases	9 (5.4)	46 (27.7)	33 (19.9)	78 (47.0)	166 (100)
Sex					
Male	3 (3.5)	23 (27.1)	21 (24.7)	38 (44.7)	85 (51.2)
Female	6 (7.4)	23 (28.4)	12 (14.8)	40 (49.4)	81 (48.8)
Placement					
Hospital	2 (20.0)	4 (40.0)	1 (10.0)	3 (30.0)	10 (6.0)
Home	7 (4.5)	42 (26.9)	32 (20.5)	75 (48.1)	156 (94.0)
Symptoms					
Asymptomatic	4 (3.8)	26 (25.0)	24 (23.1)	50 (48.1)	104 (62.6)
Mild	0	12 (32.4)	8 (21.6)	17 (46.0)	37 (22.3)
Moderate	5 (22.7)	6 (27.3)	1 (4.5)	10 (45.5)	22 (13.3)
Severe	0	2 (66.7)	0	1 (33.3)	3 (1.8)
Chronic disease					
Yes	0	3 (33.3)	2 (22.2)	4 (44.5)	9 (5.4)
No	9 (5.7)	43 (27.4)	31 (19.7)	74 (47.2)	157 (94.6)

N, number.
